# Worldwide Phylogenetic Group Patterns of *Escherichia coli* from Commensal Human and Wastewater Treatment Plant Isolates

**DOI:** 10.3389/fmicb.2017.02512

**Published:** 2017-12-21

**Authors:** Nancy de Castro Stoppe, Juliana S. Silva, Camila Carlos, Maria I. Z. Sato, Antonio M. Saraiva, Laura M. M. Ottoboni, Tatiana T. Torres

**Affiliations:** ^1^Centro de Biologia Molecular e Engenharia Genética, Universidade Estadual de Campinas, Campinas, Brazil; ^2^Núcleo de Pesquisa em Biodiversidade e Computação (BioComp-USP)-Universidade de São Paulo, São Paulo, Brazil; ^3^Secretaria de Estado de Saúde de Mato Grosso, Cuiabá, Brazil; ^4^Departamento de Genética e Biologia Evolutiva, Universidade de São Paulo, São Paulo, Brazil; ^5^Departamento de Análises Ambientais, Companhia Ambiental do Estado de São Paulo-CETESB, São Paulo, Brazil; ^6^Departamento de Engenharia de Computação e Sistemas Digitais, Escola Politécnica da USP, São Paulo, Brazil

**Keywords:** phylogenetic groups, *Escherichia coli*, multivariate analysis, wastewater, commensal strains

## Abstract

*Escherichia coli* is an important microorganism in the gastrointestinal tract of warm-blooded animals. Commensal populations of *E. coli* consist of stable genetic isolates, which means that each individual has only one phylogenetic group (phylogroup). We evaluated the frequency of human commensal *E. coli* phylogroups from 116 people and observed that the majority of isolates belonged to group A. We also evaluated the frequency of phylogroups in wastewater samples and found a strong positive correlation between the phylogroup distribution in wastewater and human hosts. In order to find out if some factors, such as geographical location, and climate could influence the worldwide phylogroup distribution, we performed a meta-analysis of 39 different studies and 24 countries, including different climates, living areas, and feeding habits. Unexpectedly, our results showed no substructuring patterns of phylogroups; indicating there was no correlation between phylogroup distribution and geographic location, climate, living area, feeding habits, or date of collection.

## Introduction

*Escherichia coli* is a facultative anaerobic microorganism found in the gastrointestinal tract of warm-blooded animals, with which it maintains a mostly symbiotic relationship. *E. coli* has also been found in soil, water, and sediments not directly influenced by sewage discharges. Besides commensal strains, there exist also pathogenic variants of *E. coli*, capable of causing either intestinal or extraintestinal diseases. Pathogenic strains were probably derived from commensal strains following the horizontal acquisition of chromosomal and extrachromosomal genes and operons, as well as gene loss (Tallon et al., 2005; Whitman et al., 2006; Tenaillon et al., 2010). The pangenome structure of *E. coli*, which includes pathogenic and commensal isolates, comprises a core of 2,200 genes, a broad range of unique genes, and a reservoir with more than 13,000 genes, indicating that these bacteria have a high potential for diversity and pathogenesis (Rasko et al., 2008). *E. coli* populations within a host are shaped by multiple host and environmental factors. In spite of recombination events, these populations are commonly described as having a clonal structure (Tenaillon et al., 2010), as demonstrated by multilocus enzyme electrophoresis, serotyping, biotyping, random amplified polymorphic DNA, and restriction fragment length polymorphism (Selander and Levin, 1980; Caugant et al., 1985; Miller and Hartl, 1986; Desjardins et al., 1995). This clonal character derives from the fact that, at any given time, each person has a predominant strain that constitutes more than half of the isolated colonies. Intra-host diversity nevertheless varies among human populations. Analysis of human commensal *E. coli* from different climate regions showed that subjects in tropical areas exhibited more diverse *E. coli* microbiota than those in temperate areas (Escobar-Páramo et al., 2004). A number of host and environmental factors influence inter-host diversity. Many *E. coli* clones have a broad geographical distribution (Ochman and Selander, 1984) and are shared by hosts of different species. Although the abundance of different groups varies according to species, four phylogenetic groups are predominant in several human and animal populations (Clermont et al., 2000). These main groups (A, B1, B2, and D) were firstly identified by multilocus enzyme electrophoresis, and were also recovered by multilocus sequence typing using 2.6 million nucleotides of the *E. coli* genome (reviewed in Tenaillon et al., 2010), indicating that these groups are genetic entities. Despite not being necessarily monophyletic, the similarity of the results obtained by enzyme electrophoresis and sequence typing, illustrates that these groups are still useful to cluster strains in a relevant way (Tenaillon et al., 2010).

Among the tools for studying *E. coli* population genetics, phylogrouping triplex PCR has been widely applied owing to its simplicity and rapidity (Clermont et al., 2000; Tenaillon et al., 2010). This method can quickly assign *E. coli* isolates to one of the four major phylogenetic groups (phylogroup): A, B1, D, or B2, which makes it useful for population genetics, classification of extraintestinal pathogenic and commensal strains, and host-source relationships (Duriez et al., 2001; Escobar-Páramo et al., 2004; Orsi et al., 2007; Gordon et al., 2008; Unno et al., 2009; Carlos et al., 2010; Tenaillon et al., 2010).

Human commensal strains belong mostly to group A (43%; Escobar-Páramo et al., 2006; Li et al., 2010), however, in tropical areas both groups A and B1 are prevalent (Escobar-Páramo et al., 2004). Instead, strains isolated from animals fall mostly into group B1 (34–50%; Higgins et al., 2007; Ishii et al., 2007; Carlos et al., 2010), suggesting an association between phylogenetic groups and host species. Within the same host species, geography, climate, diet, gut morphology, body mass, sex, age, hygiene level, *inter alia* may be associated with the distribution of phylogroups (Gordon and Cowling, 2003; Escobar-Páramo et al., 2006).

Bailey et al. (2010a) studied the worldwide distribution of phylogroups, but without correlating them with geographic location. Escobar-Páramo et al. (2004) hypothesized that the geographic and climatic location of human populations could have an important role in defining the *E. coli* phylogroup distribution. These authors observed that the French population was distinct from the American and the African ones and the 10 different locations were separated in two clusters, tropical and temperate zones. However, no formal tests were performed to study the factors underlying such difference. Li et al. (2010) compared human commensal *E. coli* isolated from China with those studied by Escobar-Páramo et al. (2004) and observed that, even though China belonged to the temperate belt, it shared a phylogroup distribution similar to that found in the tropics. No further climate classification was used in these studies. Tenaillon et al. (2010) compiled the prevalence of *E. coli* groups in humans and reported a shift from A to B2 as the most frequent *E. coli* phylogroup group in France in 1980 and 2000, respectively. Additional data showed that group A was the most common phylogroup in Africa (Mali and Benin), Asia (Pakistan), Europe (Croatia), and South America (French Guiana, Colombia, and Bolivia), while B2 was the most common phylogroup in Europe (Sweden), North America (USA), developed Asia (Japan), and Oceania (Australia). Based on these results, the authors suggested that socioeconomic factors rather than geographic location or climate could determine the phylogroup distribution.

Another way for studying the distribution of *E. coli* strains is using wastewater isolates as surrogates for human commensal *E. coli*. Isolates from wastewater can be treated as a pool of clones derived from a local human population, thus reducing the sampling effort. Furthermore, there is no need for approval of the study by an ethics committee. For these reasons, *E. coli* isolates from wastewater have been used extensively to study the distribution of phylogroups (USEPA, 2005; Duran et al., 2006; Jiang et al., 2007; Kaneene et al., 2007; Ahmed et al., 2009; Fremaux et al., 2009; Silkie and Nelson, 2009; Kelty et al., 2012). Phylogroups D and B2 were predominant among *E. coli* isolated from wastewater samples, regardless of the climatic zone, accounting for 50% of isolates in a temperate area and more than 95% in a subtropical one (Anastasi et al., 2010; Mokracka et al., 2011). These phylogroups are frequently associated with pathogenic strains of *E. coli* (Clermont et al., 2000). Hence, Anastasi et al. (2010) suggest the use of phylogroup as a simple tool to determine if strains are pathogenic or not.

Although dispersal limitation and selection are the main processes influencing the distribution of free-living microbes, the host microenvironment also plays a major role in defining the population structure of commensal isolates. Here, we tested for the presence of discontinuity against the null hypothesis, in which the distribution of phylogenetic groups was limited by dispersal across space.

Differences in phylogroup distribution have been observed in many independent studies, and these differences were attributed to climate, feeding habits, geographic location, *inter alia*. However, no formal statistical framework was used to test the effect of these parameters in phylogroup distribution. To fill this gap, we compared the phylogenetic distribution of *E. coli* strains isolated from human and wastewater samples. Furthermore, we performed a meta-analysis of the worldwide phylogroup distribution from human *E. coli* isolates, and looked for patterns of association between the commensal isolates and host geographic location, climate, host feeding habits, or host living area, indicating local adaptation. We also tested if collection date could explain the distribution of phylogroups.

## Materials and methods

### *E. coli* isolation from human feces

Feces from 116 adult humans were collected using sterile swabs and Cary-Blair transport medium. They were streaked on Endo agar LES (Difco) and incubated for 24 h at 35°C. Three lactose-positive colonies (pink to dark-red with a metallic surface sheen) were picked from each sample and tested for citrate utilization, lactose fermentation, oxidase activity, l-lysine decarboxylase activity, motility, glucose and sucrose fermentation, tryptophan deamination, indole production, urea hydrolysis, and sulfide production. One typical *E. coli* profile strain from each individual host was re-isolated on nutrient agar, incubated for 24 h at 35°C, and kept at −70°C in tryptic soy broth (Difco) with 10% glycerol (v/v) for further analysis (ATCC, 2017).

The Research Ethics Committee of the State University of Campinas School of Medical Sciences has approved the present study (Permission 049/09) and all participants gave their informed written consent. Human samples were collected from healthy individuals, representing equal numbers of males and females living in the São Paulo Metropolitan area. The age interval of the subjects ranged from 19 to 79 years (average = 43) and BMI (body mass index) ranged from 17.5 to 40.9 (average = 26). The majority of subjects (>89%) had omnivore feeding habits.

### *E. coli* isolation from raw wastewater

Five wastewater treatment plants (WTPs) in the São Paulo Metropolitan area (Parque Novo Mundo, Jesus Netto, Barueri, São Lourenço da Serra, and Vinhedo) were sampled two to three times. All WTPs received domestic discharges, while Parque Novo Mundo and Barueri plants received also industrial discharges. Raw wastewater samples were collected in sterile bottles according to standard methods (APHA, 2010) and samples were analyzed using the membrane filter technique according to U.S. Environmental Protection Agency method 1603 (USEPA, 2002) as previously described by Stoppe et al. (2014).

### DNA isolation and phylogenetic grouping

Genomic DNA from *E. coli* strains was isolated with the Wizard Genomic DNA Purification Kit (Promega) according manufacturer's instructions. The phylogroup of *E. coli* strains was determined by triplex PCR as previously described by Clermont et al. (2000) and Stoppe et al. (2014).

### Meta-analysis of human isolates

Meta-analysis was performed using data from this and previous studies (Table 1). Commensal *E. coli* strains isolated from healthy humans were analyzed in terms of Koppen climate classification (Peel et al., 2007), feeding habits (western or other), living area (urban or rural), and geographic distance (km).

**Table 1:** Distribution of commensal *E. coli* phylogenetic groups isolated from human feces.

**#**	**City or region, country**	Koppen climate classification^*^	**Feeding habits**	**Living area**	**Approximated geographic coordinates**		**Phylogenetic group [*n* (%)]**				**References**
							**A**	**B1**	**B2**	**D**	
1	São Paulo, Brazil	Aw	Western	Urban	23°32′S	46°38′W	56 (48.3)	6 (5.2)	19 (16.4)	35 (30.2)	This study
2	Sydney, Australia	Cfa	Western	Urban	33°52′S	151°12′E	39 (37.1)	10 (9.5)	31 (29.5)	25 (23.8)	Bailey et al., 2010a,b
3	Maroochydore, Australia	Cfa	Western	Urban	26°39′S	153°05′E	23 (15.2)	16 (10.6)	43 (28.5)	69 (45.7)	Vollmerhausen et al., 2011
4	Cotonou, Benin	Aw	Other	Urban	06°22′N	02°26′E	23 (50.0)	15 (32.6)	8 (17.4)	0 (0.0)	Escobar-Páramo et al., 2004
5	Alto de los Zarzos, Bolivia	Aw	Other	Rural	21°28′S	63°54′W	87 (77.0)	11 (10.0)	6 (5.0)	9 (8.0)	Pallechi et al., 2007
6	Villamontes, Bolivia	Aw	Other	Urban	21°15′S	63°32′W	23 (79.3)	0 (0.0)	0 (0.0)	6 (20.7)	Riccobono et al., 2012
7	São Paulo, Brazil	Aw	Western	Urban	23°32′S	46°38′W	38 (40.4)	8 (8.5)	12 (12.8)	36 (38.3)	Carlos et al., 2010
8	Calgary, Canada	Dfc	Western	NA	51°02′N	114°03′W	15 (13.9)	13 (12.0)	58 (53.7)	22 (20.4)	White et al., 2011
9	Fuzhou, China	Cfa	Other	Urban	26°04′N	119°17′E	142 (43.7)	76 (23.4)	52 (16.0)	55 (16.9)	Li et al., 2010
10	Beijing, China	Dwa	Other	Urban	39°54′N	116°24′E	11 (12.0)	0 (0.0)	44 (47.8)	37 (40.2)	Luo et al., 2011
11	Bogota, Colombia	Cfb	Western	Urban	04°35′N	74°04′W	16 (57.1)	1 (3.6)	7 (25.0)	4 (14.3)	Escobar-Páramo et al., 2004
12	Olib and Silba, Croatia	Csa	Western	NA	44°22′N	14°43′E	20 (35.1)	18 (31.6)	11 (19.3)	8 (14.0)	Duriez et al., 2001
13	Copenhagen, Denmark	Cfb	Western	NA	55°40′N	12°34′E	35 (20.5)	38 (22.2)	50 (29.2)	48 (28.1)	Damborg et al., 2009; Petersen et al., 2009; Jakobsen et al., 2010
14	Paris, France	Cfb	Western	Urban	48°51′N	02°15′E	113 (48.3)	21 (9.0)	43 (18.4)	57 (24.4)	Duriez et al., 2001; Escobar-Páramo et al., 2004; Leflon-Guibout et al., 2008
15	Brittany, France	Cfb	Western	Rural	48°12′N	02°55′W	14 (28.0)	13 (26.0)	12 (24.0)	11 (22.0)	Escobar-Páramo et al., 2004
16	Brest, France	Cfb	Western	Rural	48°23′N	04°29′W	3 (14.3)	5 (23.8)	7 (33.3)	6 (28.6)	Escobar-Páramo et al., 2004
17	Tours, France	Cfb	Western	Urban	47°23′N	00°37′E	6 (25.0)	5 (21.0)	7 (29.0)	6 (25.0)	Escobar-Páramo et al., 2004
18	Western France	Cfb	Western	Urban	48°06′N	01°41′W	12 (48.0)	3 (12.0)	5 (20.0)	5 (20.0)	Mereghetti et al., 2002
19	National Park, French Guiana	Af	Other	Rural	02°35′N	53°33′W	59 (63.4)	19 (20.4)	3 (3.2)	12 (12.9)	Escobar-Páramo et al., 2004
20	Munchengladbach, Germany	Cfb	Western	NA	48°08′N	11°34′E	16 (43.3)	10 (27.0)	6 (16.2)	5 (13.5)	Sorsa et al., 2007
21	Babylon, Iraq	BWh	Other	Urban	32°28′N	44°33′E	6 (60.0)	3 (30.0)	0 (0.0)	1 (10.0)	Abdul-Razzaq and Abdul-Lateef, 2011
22	Tokyo, Japan	Cfa	Other	Urban	35°41′N	139°41′E	30 (16.6)	17 (9.4)	91 (50.3)	43 (23.8)	Obata-Yasuoka et al., 2002; Harada et al., 2012
23	Kyoto, Japan	Cfa	Other	NA	35°00′N	135°46′E	21 (42.0)	16 (32.0)	10 (20.0)	2 (6.0)	Kanamaru et al., 2006
24	Seoul, Korea	Dwa	Other	Urban	37°34′N	126°58′E	78 (38.0)	37 (18.0)	47 (22.9)	43 (21.0)	Lee et al., 2010
25	Jeonnam Province, Korea	Dwa	Other	NA	34°52′N	126°59′E	42 (29.8)	48 (34.0)	0 (0.0)	31 (36.2)	Unno et al., 2009
26	Bay Reserve, Mali	As	Other	NA	13°00′N	05°00′E	13 (23.6)	32 (58.2)	1 (1.8)	9 (16.4)	Duriez et al., 2001
27	Oslo and Telemark, Norway	Dfb	Western	NA	59°54′N	10°45′E	6 (30.0)	5 (25.0)	3 (15.0)	6 (30.0)	Grude et al., 2007
28	Lahore, Pakistan	BSh	Other	Urban	31°32′N	74°20′E	74 (47.0)	28 (18.0)	19 (12.0)	36 (23.0)	Nowrouzian et al., 2009
29	Villa Real, Portugal	Csa	Western	NA	41°18′N	07°31′W	5 (8.6)	6 (10.3)	38 (65.5)	9 (15.5)	Silva et al., 2010
30	Angaiza, Peru	Af	Other	Rural	03°35′S	71°36′W	80 (72.0)	19 (17.0)	3 (3.0)	9 (8.0)	Bartoloni et al., 2009
31	Madrid, Spain	Csa	Western	Urban	40°25′N	03°42′W	28 (49.1)	11 (19.3)	4 (7.0)	14 (24.6)	Machado et al., 2005; Valverde et al., 2009
32	Barcelona, Spain	Csa	Western	Urban	41°23′N	02°10′E	40 (33.0)	23 (19.0)	20 (17.0)	37 (31.0)	Moreno et al., 2009
33	Gothenburg, Sweden	Cfb	Western	NA	57°42′N	11°58′E	147 (30.5)	61 (12.7)	205 (42.5)	69 (14.3)	Nowrouzian et al., 2005, 2006; Karami, 2007
34	Geneva and Ticino, Switzerland	Cfb	Western	Urban	46°05′N	07°30′E	4 (40.0)	1 (10.0)	2 (20.0)	3 (30.0)	Grasselli et al., 2009
35	Boise, United States	BSk	Western	Rural	43°33′N	116°22′W	32 (26.2)	17 (13.9)	35 (28.7)	38 (31.1)	Hannah et al., 2009
36	Minneapolis, United States	Dfb	Western	Urban	44°59′N	93°22′W	20 (13.6)	22 (15.0)	78 (53.1)	27 (18.4)	Zhang et al., 2002; Sannes et al., 2004; Johnson et al., 2005; Logue et al., 2012
37	Ann Arbor, United States	Dfa	Western	Urban	42°14′N	83°44′W	18 (20.5)	11 (12.5)	42 (47.7)	17 (19.3)	Zhang et al., 2002
38	Fargo, United States	Dfb	Western	Urban	46°52′N	96°47′W	33 (16.2)	32 (15.7)	110 (53.9)	29 (14.2)	Logue et al., 2012
	Total						1,428	677	1,132	879	

* *Peel et al., 2007. NA, not available*.

**Figure 1:**
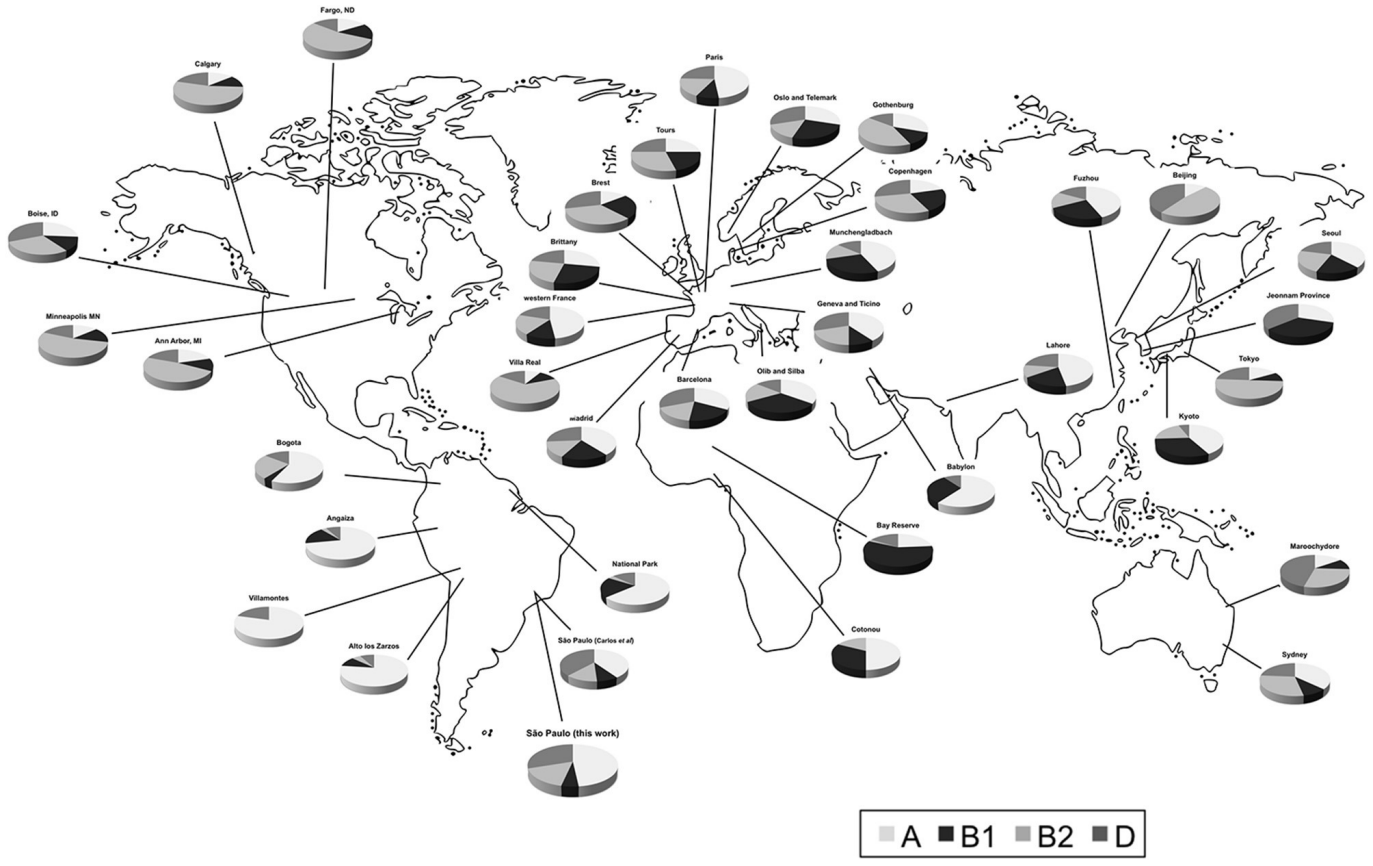
Distribution of human commensal *E. coli* phylogenetic groups in selected studies from different sites.

Data were selected according to the following criteria: (1) *E. coli* were isolated from feces of healthy individuals (both sexes); (2) one isolate per individual or representative strain was obtained; (3) the phylogroup classification was done according to Clermont et al. (2000); (4) approximate geographic location (city or region) where the strains were obtained was recorded. In some cases, the same datasets were analyzed in two or more studies. In the present evaluation, we exerted care not to include the same datasets twice. We combined data of isolates from the same city or region (Table 1 and Figure 1). We also used geographic coordinates and local climate to identify patterns in phylogroup distribution.

#### Visualization of data distribution and correlation analysis

Visualization of phylogroup distribution was achieved by means of a notched boxplot using the “*boxplot*” function from the “*graphics*” package in R (R Development Core Team, 2012a).

Next, we applied the “*pairs*” function from the same package with the following arguments: (1) “*upper.panel*,” to apply correlation analysis between variables; (2) “*diag*.*panel*,” to represent the frequency of distribution of the data using histograms; and (3) “*lower.panel*,” to smooth the data.

#### Analysis of variance (ANOVA)

ANOVA was performed to test differences in phylogroup distribution with different response variables (city, country, Koppen climate classification, feeding habits, and living area). To this end, we used the “*aov*” function from the “*stats*” package in R (R Development Core Team, 2012b).

The *post-hoc* Tukey HSD test was used to identify significant differences between means using the function “*TukeyHSD*” from the “*stats*” package.

#### Clustering and correspondence analysis (CA)

The phylogroup distribution of the different sites was clustered hierarchically using R. We calculated the Euclidean distances by using the function “dist,” and clustered the data points by using the function “htclust” testing two clustering methods (method = “complete,” and method = “median”). Both functions, “dist” and “htclust,” are part of the “*stats*” package. The dendrograms were plotted using the function “*plot*” from the “*graphics*” package.

To determine the existence of a correspondence between phylogroups and geographic location CA was performed using the packages “*ca”* (Nenadic and Greenacre, 2007), “*FactoMineR”* (Lê et al., 2008), and “*vcd”* (Meyer et al., 2014) in R. The agglomerative hierarchical clustering was done on the results of the factor analysis. To cluster the data and determine the optimal level of division, we used the method implemented in the R package “FactoMineR” (Lê et al., 2008). The function first built a hierarchical tree. Then the sum of the within-cluster inertia is calculated for each partition. The suggested partition is the one with the higher relative loss of inertia (Husson et al., 2010). The Ward criterion has to be used in the hierarchical clustering because it is based on the multidimensional variance (inertia). Locations (rows) were clustered using metric = “euclidean,” and method = “ward,” in the function HCPC. This allowed us to test whether phylogroups were could be used to group locations in meaningful clusters.

#### Probability distribution model: poisson and gamma distributions

To calculate mean and variance for the Poisson test, we used the *ddply* function (Wickham, 2011) from the “*plyr*” package in R.

Given that the probability distribution model of phylogroup data was not known a priori, a more general probability model distribution, such as gamma, was used. To this end, we applied the parameters “*shape*” and “*rate*,” and the maximum-likelihood estimation (MLE) “*fitdistr*” function from the “*mass*” package in R (Ripley et al., 2013). Next, we generated a sample theoretical gamma distribution (cumulative distribution function, CDF) for the parameters “*shape*” and “*rate*” using the “*pgamma*” function from the “*stats*” package. Finally, to test the similarity between each phylogroup data distribution (empirical CDF) and a theoretical gamma distribution (gamma CDF), the Kolmogorov–Smirnov test (K–S test) was performed, using the “*ks.test*” function from the “*stats*” package.

#### Identification of patterns using social network analysis *w-Clique* metric

The *w-clique* metric was used to identify cohesive subgroups (clusters) in the network (Araújo et al., 2008). As described by Stoppe et al. (2014) and Silva et al. (2014), the *Dieta* program was first used to verify the nodes connected by strong interactions. These were encoded by a binary matrix (0/1), in which cells containing the number 1 represented interactions whose weights were higher than the average network weight (*w-cliques*; Araújo et al., 2008). Next, the *Pajek* program was used to transform the network from arcs to edges (Batagelj and Mrvar, 1998). The resulting matrix was analyzed using the *Ucinet* program (Borgatti et al., 2002) to identify the *w-cliques*.

#### Data mining classification

The “*decision tree*” algorithm (Witten et al., 2011) was used for data mining classification. To build the regression tree, we used the package “*rpart*” (Therneau et al., 2013) in R. To minimize the “*misclassification error*”—the percentage of data that the tree does not classify properly—we used different values for data training and testing.

#### Multivariate analysis

Statistical analysis was based on two multivariate analysis methods: Mantel test (Oksanen, 2013) and clustering visualizations of multidimensional data (Hurley, 2004).

The former was used to analyze the correlation between two dissimilarity matrices, based on Pearson's product-moment correlation. The dissimilarity matrices were calculated with the “*vegdist*” function, using the community ecology package “*vegan*” in R (Oksanen, 2013), with the following coefficients as parameters: Bray–Curtis (data abundance) and Jaccard (data presence/absence; Legendre and Legendre, 2012).

For the latter, the dissimilarity matrices of the phylogroup distribution (raw data) were built using the Bray–Curtis coefficient (Legendre and Legendre, 2012). The similarity matrices were prepared as a complement to the dissimilarity matrices [1-vegdist (matrix, “Bray”)].

The permutation of variables (hierarchical clustering order) was based on the classification of data, such as Koppen climate classification, feeding habits, living area, geographic distance, and collection date. This was presented graphically by the scatterplot matrix, which is commonly used for displaying multivariate data.

## Results

### Phylogroup distribution in human and wastewater isolates

Briefly, 116 strains were isolated from different individuals (male and female adults with western feeding habits and average BMI of 26). Of these strains, 48.3% belonged to group A, 5.2% to group B1, 16.4% to group B2, and 30.2% to group D (Table 1). The phylogroup distribution did not differ significantly in terms of gender, age, or BMI.

Additional 150 strains were isolated from raw wastewater samples. Of these, 44% belonged to group A, 39% to group D, 9% to group B2 and 8% to group B1.

The phylogroup distribution was similar between wastewater and human samples; groups A and D were predominant and the phylogroup distribution was confirmed by a positive correlation (Mantel test, *r* = 0.607, *P* = 0.046).

### Comparison of worldwide phylogroup distribution in humans and wastewater

To obtain a more complete picture of the phylogroup distribution worldwide, we looked at published studies on WTP and human commensal isolates from Australia, Portugal, Spain, and the United States (Zhang et al., 2002; Sannes et al., 2004; Johnson et al., 2005; Machado et al., 2005; Boczek et al., 2006; Sabaté et al., 2008; Hannah et al., 2009; Moreno et al., 2009; Valverde et al., 2009; Anastasi et al., 2010; Bailey et al., 2010a,b; Figueira et al., 2011; Vollmerhausen et al., 2011; Logue et al., 2012; Silva et al., 2012). Contrary to our present findings, no significant correlation between phylogroup distribution in humans and wastewater was reported in Australia (Mantel test, *r* = 0.003, *P* = 0.454), Portugal (Mantel test, *r* = −0.396, *P* = 0.600), Spain (Mantel test, *r* = −0.023, *P* = 0.575), and the United States (Mantel test, *r* = 0.070, *P* = 0.467).

### Meta-analysis of human isolates

Next, we compared our distribution of *E. coli* phylogroups from commensal isolates with those from other worldwide studies.

**Figure 2:**
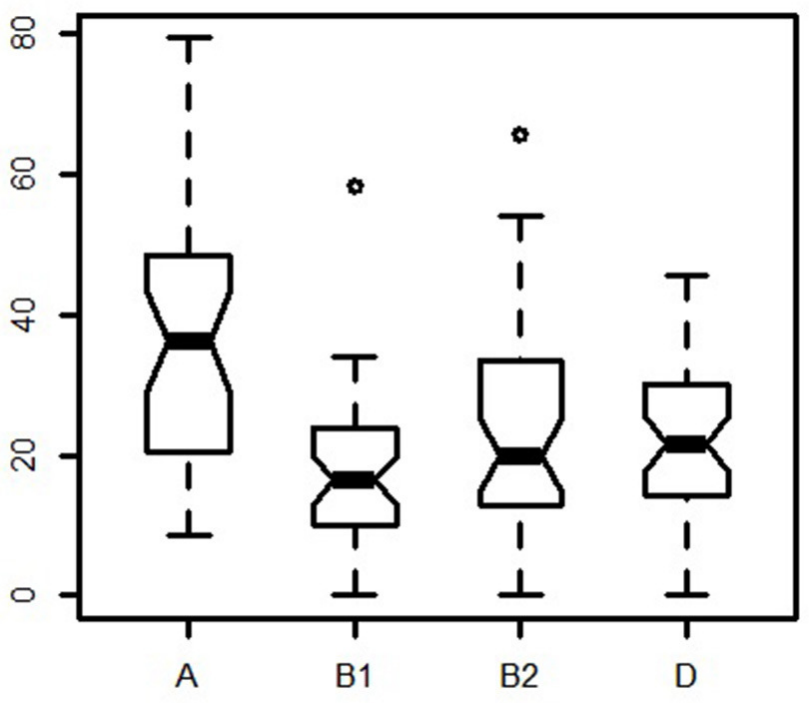
Notched boxplot of meta-analysis phylogroup distribution.

Worldwide, group A was the most common type with a median of 36.1%. It was followed by D (median 21.5%), B2 (median 20%), and B1 (median 16.4%). The widest frequency range was observed for group A (from 8.7 to 79.3%), while B2 ranged from 0 to 53.9%, D from 0 to 45.7%, and B1 from 0 to 34%. Frequency differences across locations were highest for groups A and B1, and lowest for groups B2 and D (Figure 2).

**Figure 3:**
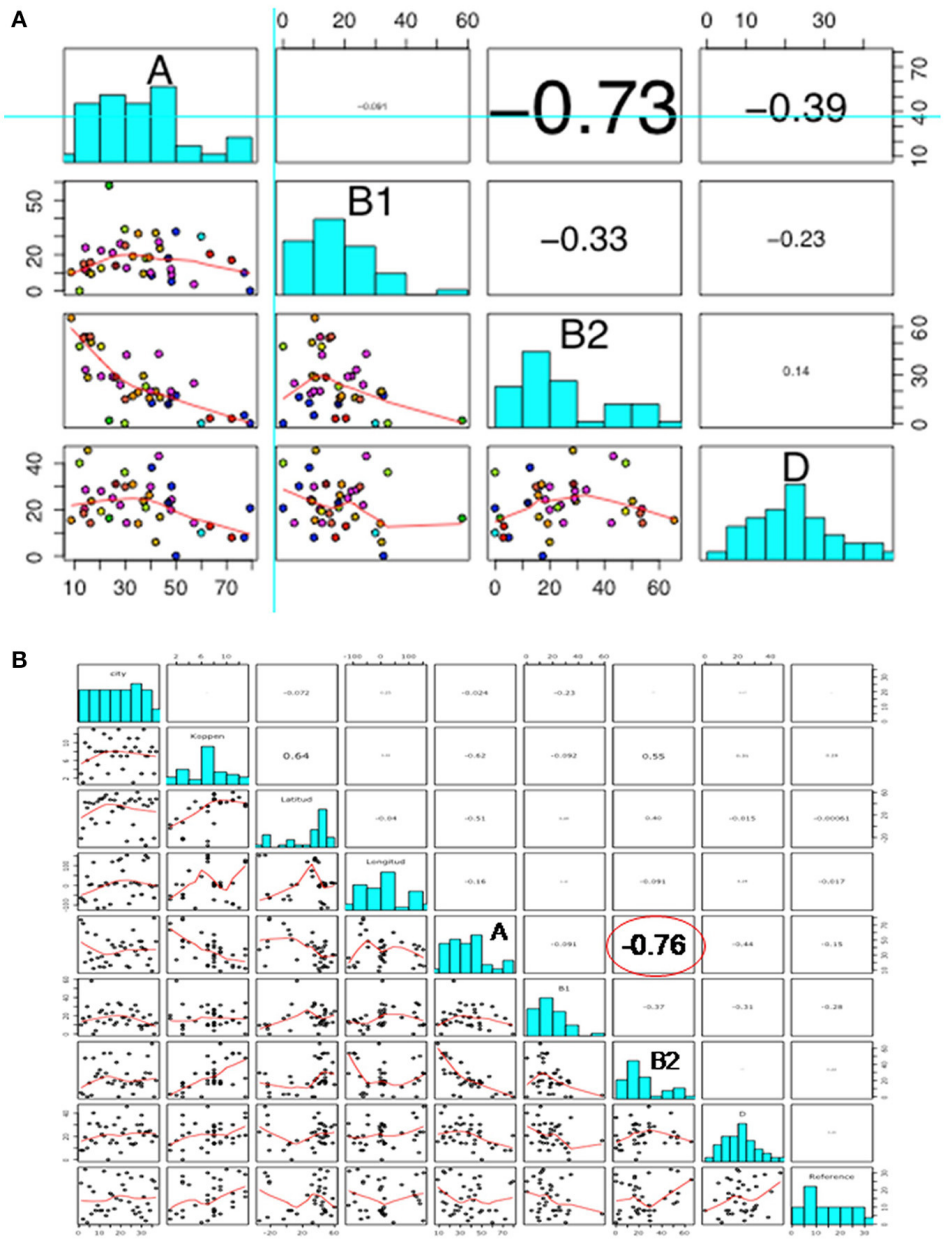
Scatterplot showing **(A)** correlation among phylogroups; and **(B)** correlation among phylogroups, feeding habits, living area, and geographical location. In both scatterplots the figures are divided in three parts, representing: (1) upper panel, correlation analysis between variables; (2) diagonal panel, histogram of the phylogroup distribution data; and (3) lower panel, the smooth function of the data.

A strong negative correlation was observed between groups A and B2 (*r* = −0.73), but not among other phylogroups (Figure 3A). When new variables (feeding habits, living area, or geographic location) were taken into account, the correlation among phylogroups did not change, suggesting that these other variables were independent (Figure 3B).

A comparison of phylogroup distributions between countries did not reveal any significant difference, except for groups A (ANOVA, *F* = 2.83, *P* = 0.05), B1 (ANOVA, *F* = 2.39, *P* = 0.05), and B2 (ANOVA, *F* = 3.14, *P* = 0.05). A Tukey HSD analysis of pairwise differences showed that Bolivia was significantly different from Portugal and the United States for both phylogroup A (respectively, *P* = 0.04 and *P* = 0.01) and phylogroup B2 (respectively, *P* = 0.04 and *P* = 0.05). For group B1, Mali was significantly different from Australia (*P* = 0.03), Bolivia (*P* = 0.01), Brazil (*P* = 0.02), China (*P* = 0.03), Colombia (*P* = 0.03), France (*P* = 0.05), and the United States (*P* = 0.03). Upon comparing the phylogroup distribution among the different climates (Koppen classification), only group A (ANOVA, *F* = 0.009, *P* = 0.01), tropical rain forest, and warm summer continental climates appeared significantly different (*P* = 0.05) from the other climates. However, following a less discriminative Koppen classification, with more countries for each climate type, the frequencies of both groups A and B2 differed significantly (*F* = 0.002, *P* = 0.01) in relation to climate. The phylogroup distribution in a tropical climate was significantly different from a temperate (*P* = 0.02) and a continental one (*P* = 0.001), irrespective of precipitation and season. Finally, the phylogroup distribution was comparable between the two different living areas, rural and urban, but differed significantly between groups A, B2, and D in terms of feeding habits, i.e., western or other diet.

**Figure 4:**
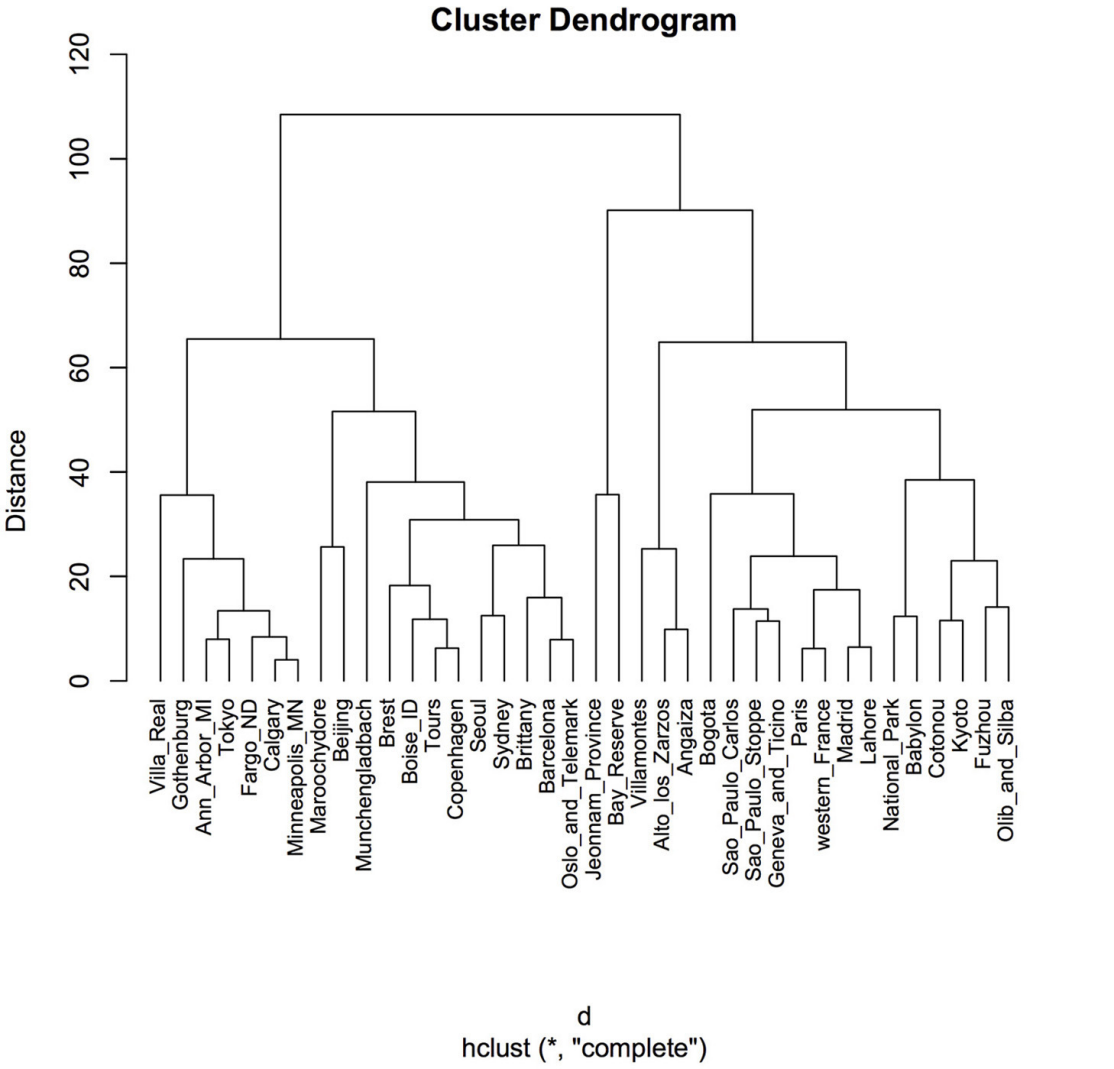
Cluster dendrogram using Euclidean distance and hierarchical cluster analysis. Distances were calculated by using the function “dist” in the R package. A hierarchical cluster analysis was done using the set of dissimilarities and the function “hclust,” with the “complete” linkage method. Using this method, when a cluster is formed, its distance to other objects is computed as the maximum distance between any object in the cluster and the other object. Iteratively, the algorithm joins the two most similar clusters until there is just a single cluster. At each iteration, distances between clusters are recomputed. “Distance” displayed in the y-axis is the value of the recomputed distance metric between clusters.

Clustering according to Euclidean distance and the “complete method” of clustering showed two major groups that did not reflect geographic proximity, feeding habit, climate, or living area (Figure 4). The same pattern was observed when the method of clustering was “median” (data not shown).

A contingency table listing cities and phylogroups indicated a strong dependency, with a correlation coefficient of 0.526 (above the 0.2 threshold). Furthermore, the chi-square score was equal to 265.42 (degrees of freedom = 20), which was highly significant (well below alpha 0.01), suggesting geographic location might be a factor in phylogroup distribution.

**Figure 5:**
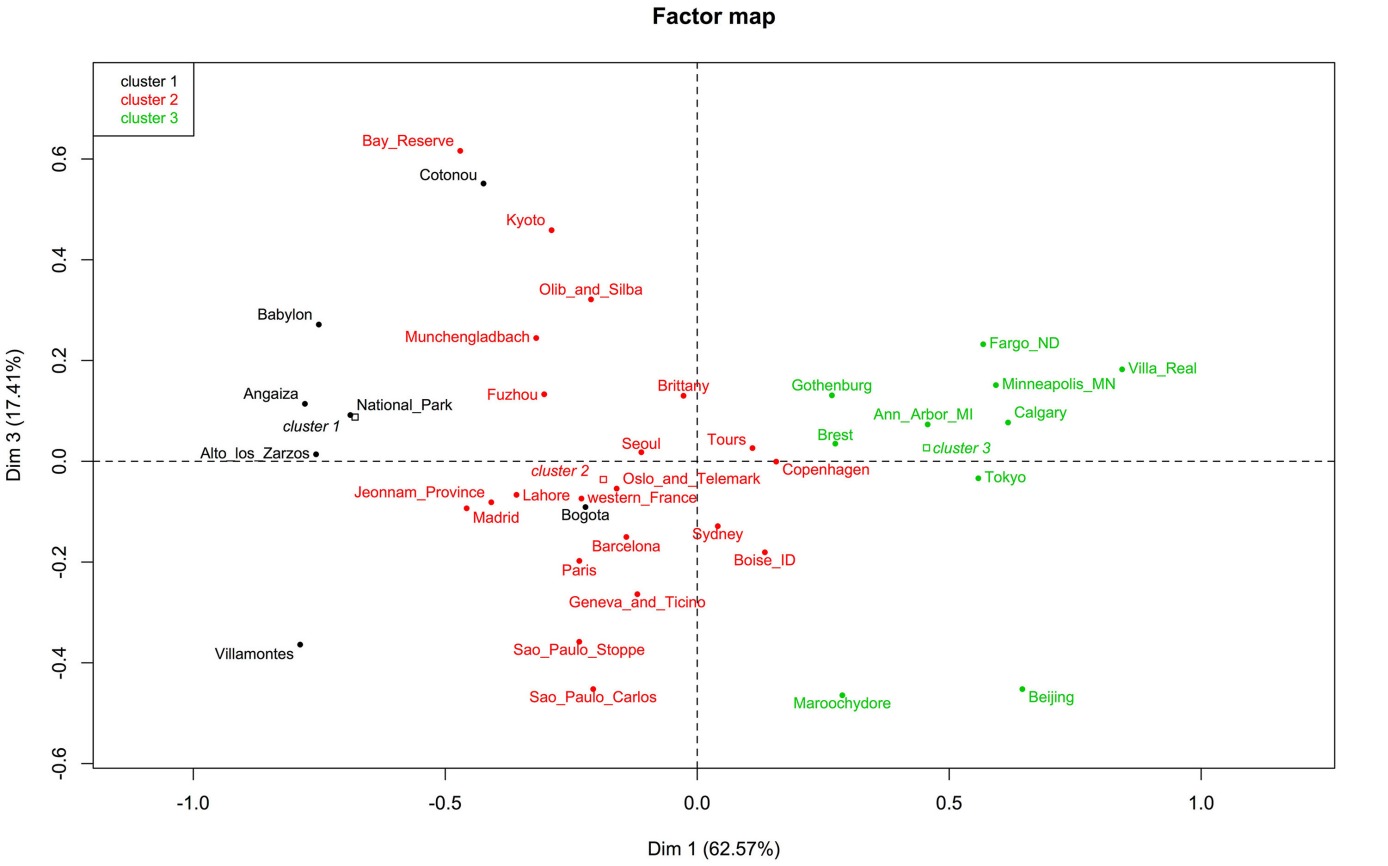
Factor map showing three clusters.

**Figure 6:**
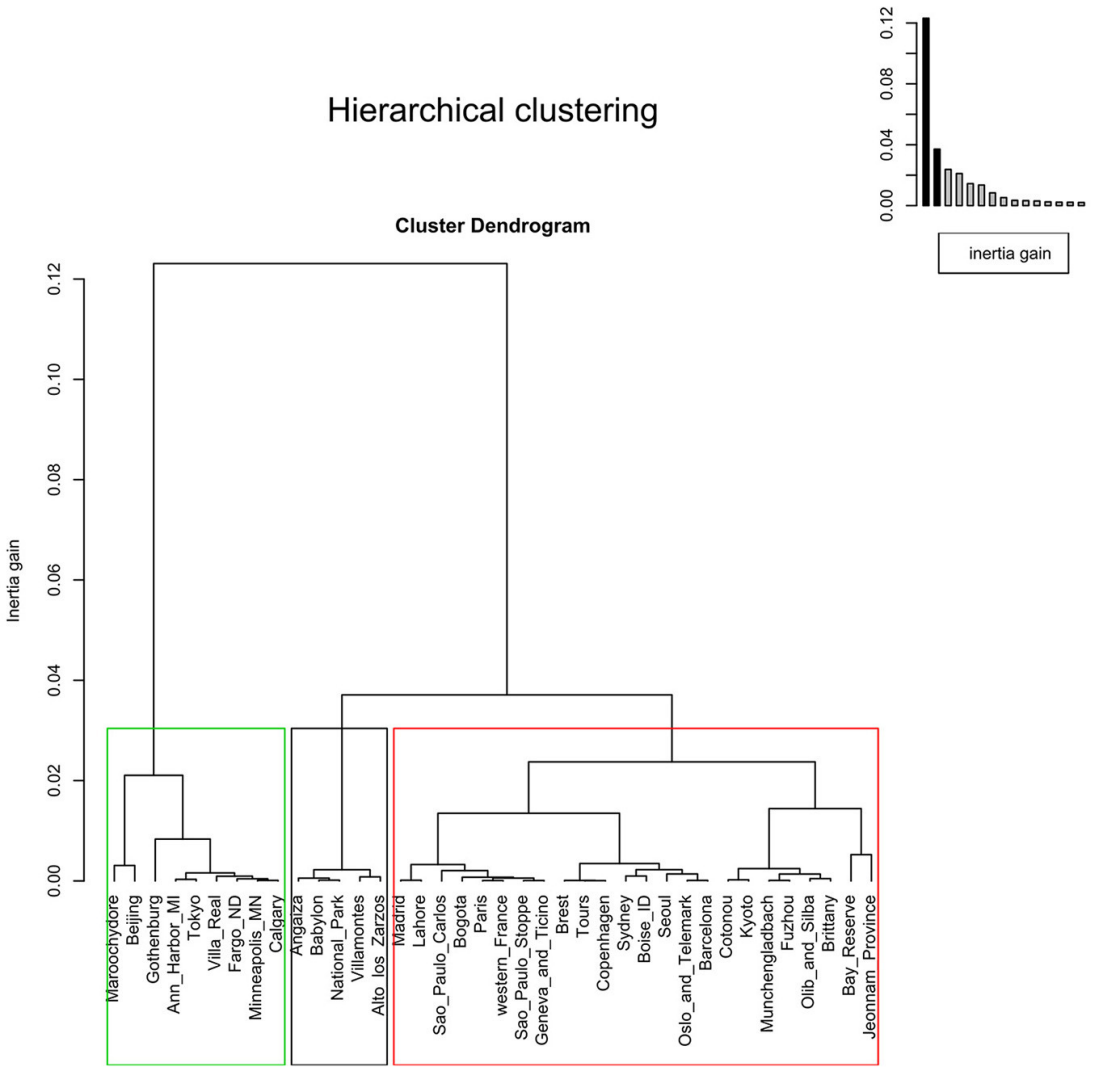
Dendrogram showing three clusters with threshold of 0.03. The agglomerative hierarchical clustering was done on the results of the correspondence analysis performed using the function “ca” in R. To cluster the data and determine the optimal level of division, the method implemented in the R package FactoMineR was used. Each level at which the merging of clusters occurs corresponds to the associated reduction of the table's inertia. The height of the tree (y-axis) corresponds to the inertia gain. Locations were clustered using metric=“euclidean,” and method =“ward,” using the function “HCPC,” and the optimal level of division was suggested as three clusters.

The factor map showed three clusters. The first containing Alto de los Zarzos, Angaiza, Babylon, Bogota, Cotonou, National Park, and Villamontes. Here, no clear pattern could be observed, since these sites were geographically isolated, and differed in terms of both, climate and living area. The same could be said of the other two clusters (Figure 5). The dendrogram also showed three clusters but, as reported in the factor map, no pattern was observed (Figure 6).

**Figure 7:**
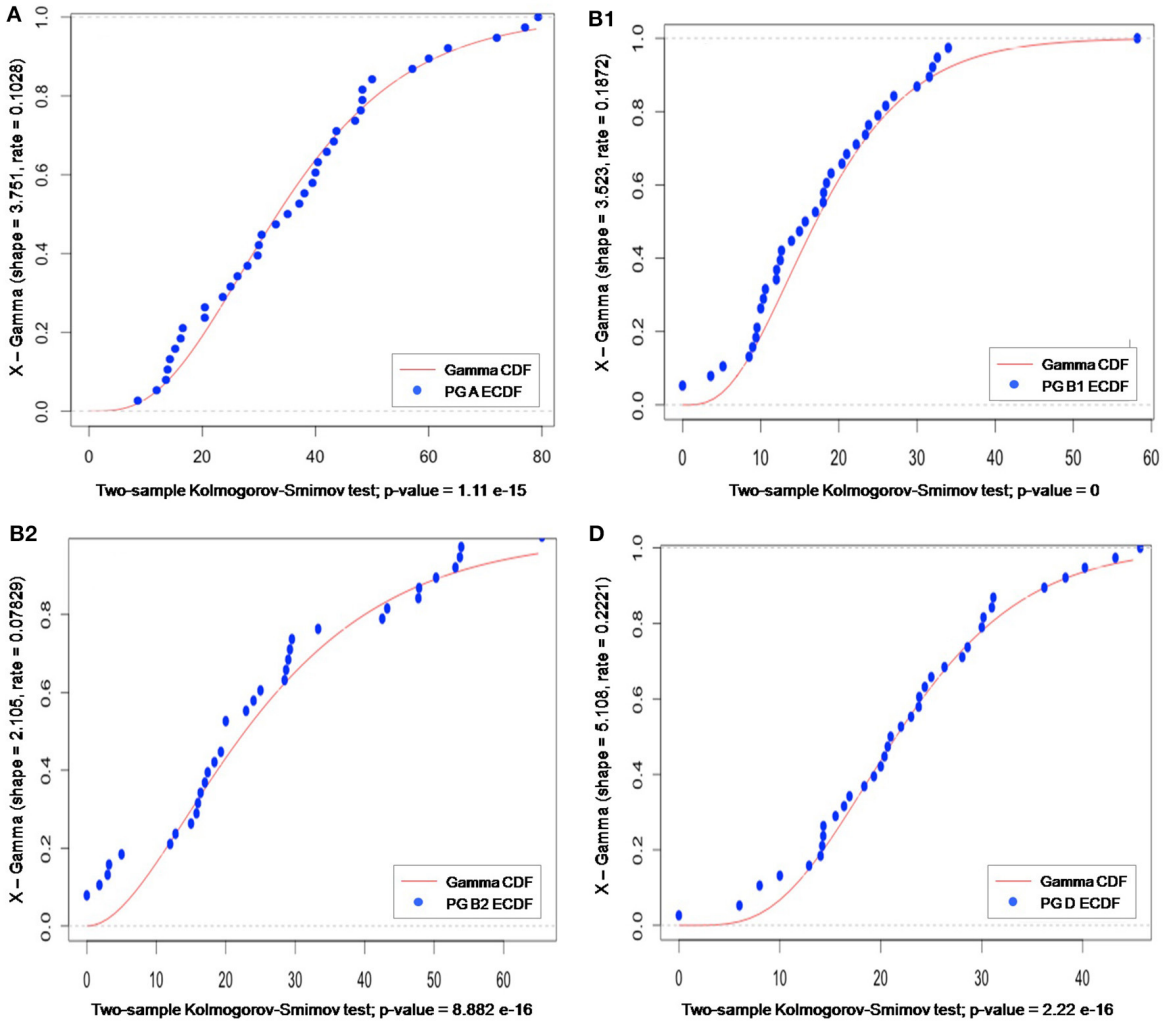
Gamma distribution of meta-analysis phylogenetic data for groups A, B1, B2, and D. The gamma cumulative distribution function (CDF) is represented by a continuous red line and the phylogroup empirical CDF by blue dots. The “*shape*” and “*rate*” parameter values, derived from the gamma distribution for a specific sample space, are given on the y-axis label; and the *p-value* (generated by K–S test) is indicated on the x-axis label.

As phylogroup data did not follow a discrete probability distribution, the use of a Poisson distribution was excluded. Instead, a gamma distribution was the probabilistic model that best fitted our data, as confirmed by calculating “*shape*” and “*rate*” parameters (using the MLE optimization algorithm) and running the K–S test (to identify the *p-value*). As gamma is a generalist continuous distribution model, it is typically used when the pattern of the data is not known (e.g., rainfall). In our case, the lower bound was zero, the upper one was not known, and no even distribution was observed around the mean, meaning that neither this model offered much information about the phylogroup data (Figure 7).

**Figure 8:**
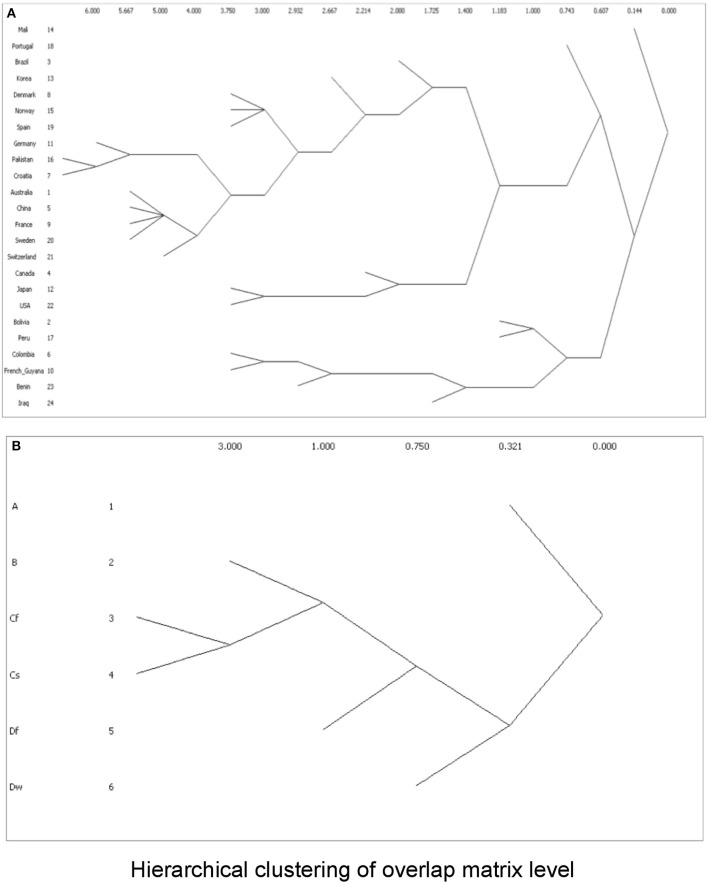
Dendrograms showing clusters according to *w-clique* metric, based on **(A)** phylogroup distribution data and countries; or **(B)** Koppen climate classification.

To cluster the phylogroup distribution according to geographic location and climate, we used *w-clique* metric. However, the clusters identified by the dendrogram did not reflect any geographic or climate classification (Figure 8). We also analyzed multiple factors simultaneously: geographic location and feeding habits; geographic location and living area; geographic location, feeding habits, and living area; climate and feeding habits; climate and living area; and climate, feeding habits, and living area. Even in this case, clusters failed to conform to any clear-cut subgroups (data not shown).

Data mining using a “*decision tree*” algorithm is a promising tool for simple classifications (Witten et al., 2011). Unfortunately, our data did not present a good level of confidence, voiding this option.

We also observed a weak correlation between geographic location and phylogroup distribution (Mantel test, *r* = 0.2059, *P* = 0.007), and moderate correlation between climate and phylogroup distribution (Mantel test, *r* = 0.399, *P* = 0.001).

**Figure 9:**
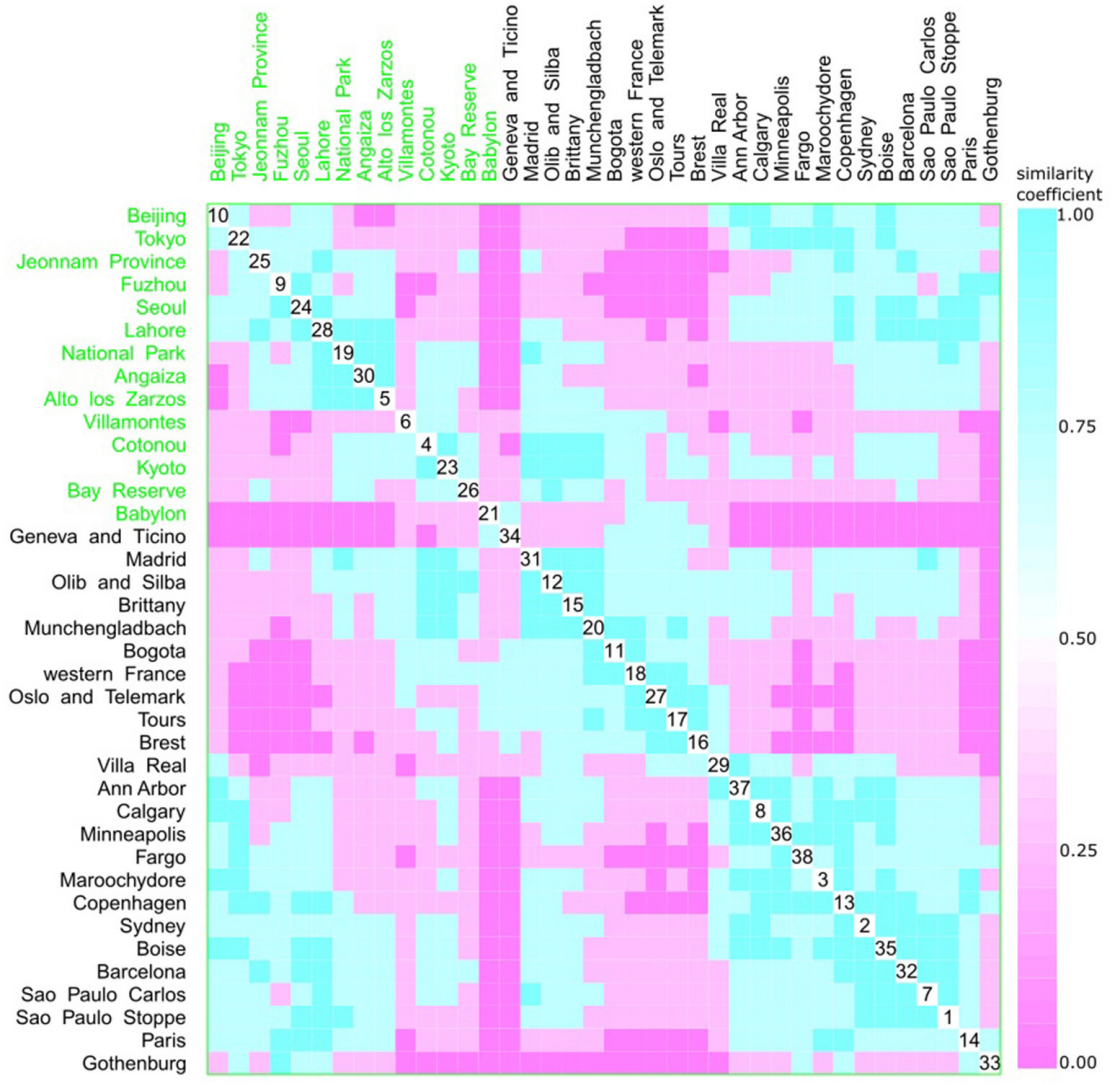
Scatterplot matrix showing multivariate analysis between phylogenetic groups and feeding habits using clustering visualization of multidimensional data (Hurley, 2004). An identical color of the scatterplot clusters denotes a similar index value; thus, according to the Bray-Curtis coefficient, data have the same pattern (“*shape*” and “*rate*”) of phylogroup distribution values. Components close to the scatterplot matrix diagonal consist of highly related variables. Three clusters can be seen, the first among cities, such as Beijing (10) and Alto de los Zarzos (5); the second is among Madrid (31) and Villa Real (29); and the last one among Villa Real (29) and Paris (33). Feeding habits: western with 24 locations, and 2529 isolates (black), and other diets with 14 locations, and 1587 isolates (green).

**Figure 10:**
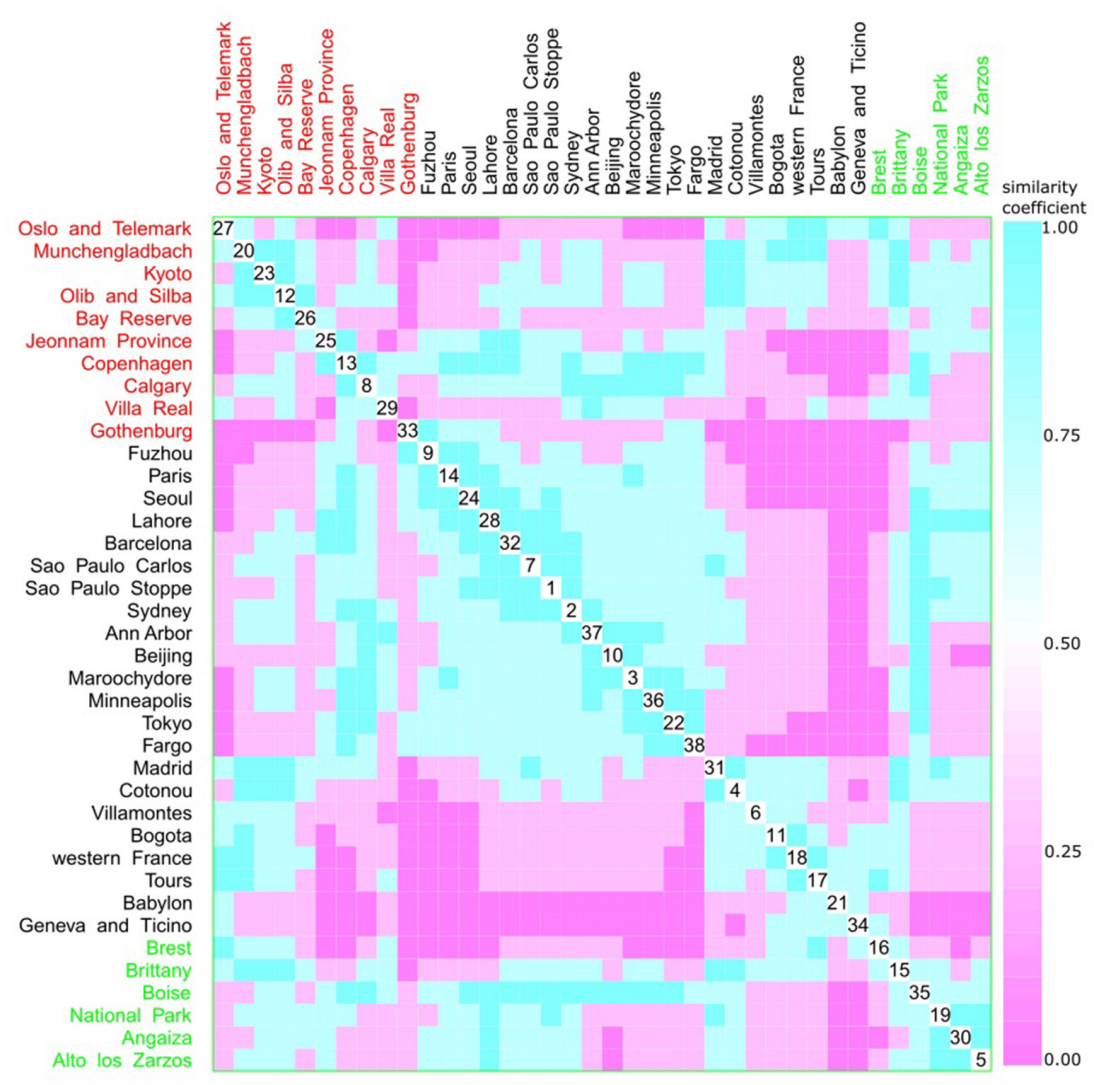
Scatterplot matrix showing multivariate analysis between phylogenetic groups and living area using clustering visualization of multidimensional data. A cluster can be seen between Gothenburg (33) and Fargo (38). Living area: urban with 22 locations, and 2,448 isolates (black), rural with 6 locations, and 510 isolates (green), and not available with 10 locations, and 1,158 isolates (red).

**Figure 11:**
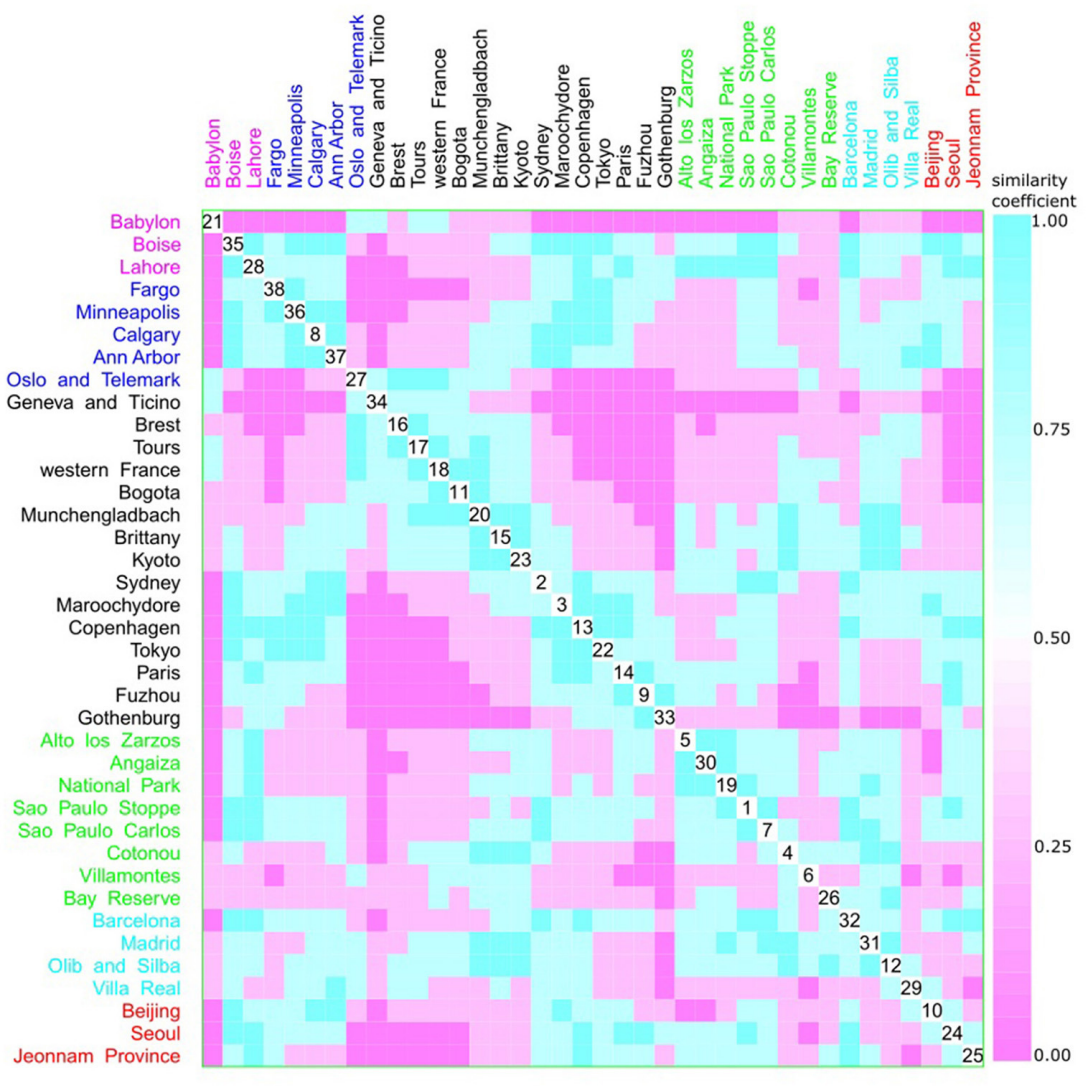
Scatterplot matrix showing multivariate analysis between phylogenetic groups and Koppen climate classification using clustering visualization of multidimensional data. Two clusters can be seen, the first among Boise (35) and Ann Harbor (37); and the second among Oslo and Telemark (27) and Gothenburg (33). Koppen climate classification: warm temperate humid subtropical with 15 locations, and 1893 isolates (black), warm temperate Mediterranean with 4 locations, and 292 isolates (cyan), equatorial with 8 locations, and 657 isolates (green), arid desert and steppe with 3 locations, and 289 isolates (magenta), continental fully humid with 5 locations, and 567 isolates (blue), and continental hot summer with 3 locations, and 418 isolates (red).

**Figure 12:**
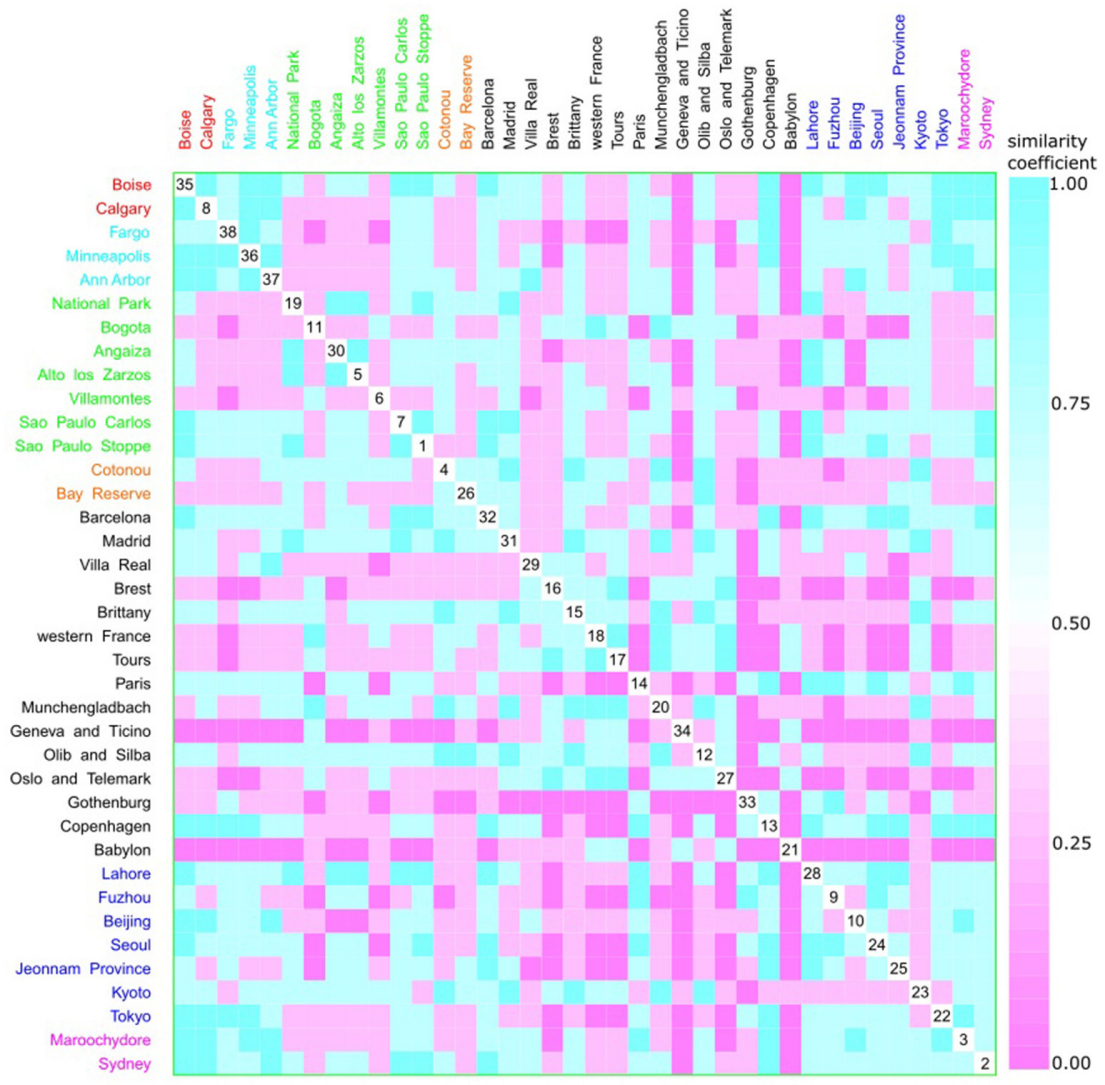
Scatterplot matrix showing multivariate analysis between phylogenetic groups and geographic distance using clustering visualization of multidimensional data. Two clusters can be seen, the first among Boise (35) and Ann Harbor (37) and the second among Tours (17) and Villa Real (29). Geographic distance based on *k*-means clustering, in which each place belongs to the cluster with the nearest mean: cluster 1 with 2 locations, and 230 isolates (red), cluster 2 with 3 locations, and 439 isolates (cyan), cluster 3 with 7 locations, and 584 isolates (green), cluster 4 with 2 locations, and 101 isolates (orange), cluster 5 with 15 locations, and 1376 isolates (black), cluster 6 with 7 locations, and 1130 isolates (blue), and cluster 7 with 2 locations, and 256 isolates (magenta).

**Figure 13:**
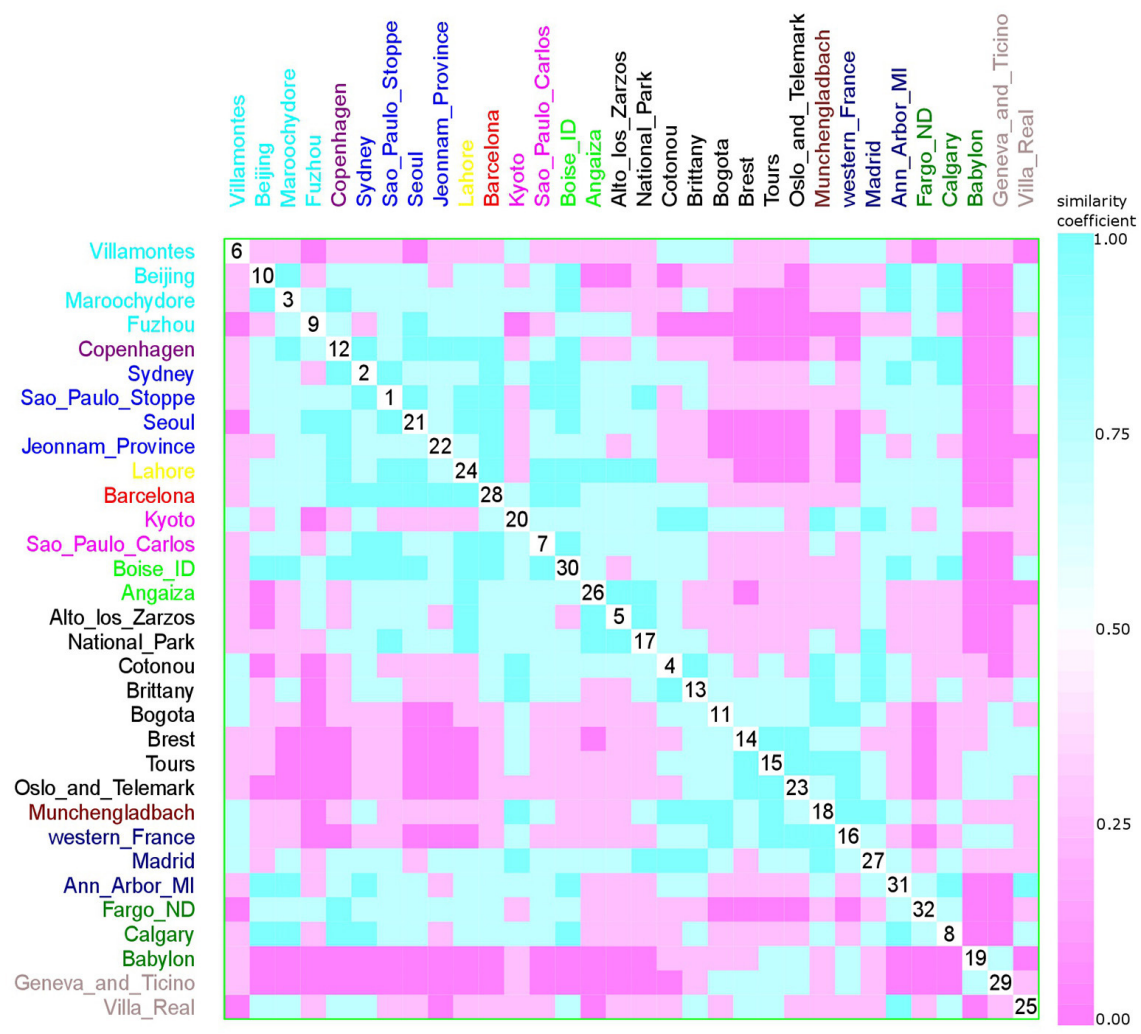
Scatterplot matrix showing multivariate analysis between phylogenetic groups and collection date using clustering visualization of multidimensional data. Year of *E. coli* isolation: 1984 with 1 location, and 157 isolates (yellow), 1994 with 2 locations, and 144 isolates (magenta), 1999 with 8 locations, and 395 isolates (black), 2001 with 3 locations, and 170 isolates (dark blue), 2002 with 2 locations, and 233 isolates (green), 2003 with 1 location, and 120 isolates (red), 2004 with 1 location, and 171 isolates (violet), 2006 with 1 location, and 37 isolates (brown), 2007 with 2 locations, and 68 isolates (gray), 2008 with 4 locations, and 567 isolates (blue), 2009 with 4 locations, and 597 isolates (cyan), and 2010 with 3 locations, and 322 isolates (dark green). Samples that were collected across a large time frame were excluded from the analysis (Paris, Gothenburg, Olib e Silba, Bay Reserve, Tokyo, and Minneapolis).

A multivariate analysis using the multidimensional data method showed an unclear relationship between phylogroups and feeding habits, living area, Koppen climate groups, and geographic distance. Accordingly, three clusters could be observed between phylogroup and feeding habits, but each one consisted of both a western and other diet (Figure 9). The same was observed for living area, where one cluster contained cities from either urban or rural areas (Figure 10), for Koppen climate groups (Figure 11), geographic distance (Figure 12), and collection date (Figure 13).

Hurley (2004) proposed that the use of categorical variables with few levels could bias the results, resulting in inaccurate clusters. As feeding habits and living areas have only two classes each, western and other diets, and urban and rural areas respectively, this might explain the observed results (Figures 9, 10). However, Koppen climate, and geographic distance comprise several categories and, despite that, only micro-clusters were observed (Figures 11, 12), indicating that the human commensal *E. coli* phylogroup distribution did not correlate with either climate or geographic distance.

## Discussion

The methods described here were used independently for a number of other biological studies. For instance, triplex PCR (the Clermont method) was used to test whether phylogenetic group frequencies varied with the age and sex of hosts (Gordon et al., 2005). Johnson et al. (2005) used this technique to differentiate commensal and pathogenic *E. coli* strains. It was also used for the identification of the sources of fecal contamination (Carlos et al., 2010). For the statistical analysis, we used mainstream tools for exploratory data analysis. Some of the tests we applied are new or not widely used, but they also have examples of applications in the literature. For example, Tanner and Jackson (2012) and Stanley and Dunbar (2013) used Social Network Analysis (SNA) to study social organization in different species. We proposed a simple classification of polluted and unpolluted sites by using SNA metrics (Stoppe et al., 2014). Buttigieg and Ramette (2014) established a web-based resource to evaluate interaction between environmental factors and microorganisms in microbial ecology studies using multivariate analyses. To our knowledge, our manuscript is the first report of the use of the combined methods to test the worldwide distribution of *E. coli* subgroups. Previous research has shown that phylogroup distribution varied across locations. The observed differences were attributed to differences in climate, living area, and feeding habits (Escobar-Páramo et al., 2004; Gordon et al., 2005; Nowrouzian et al., 2005, 2009; Karami, 2007; Pallechi et al., 2007; Vinue et al., 2008; Bartoloni et al., 2009; Hannah et al., 2009; Bailey et al., 2010b; Li et al., 2010; Nicolas-Chanoine et al., 2013). However, no formal statistical framework was used to test the effect of these parameters in phylogroup distribution. Here, we established a novel statistical workflow to test factors influencing worldwide distribution of *E. coli* subgroups, which may be of interest amongst other research groups with similar questions.

Commensal populations of *E. coli* comprise stable genetic isolates with very low rates of recombination, leading to a clonal population structure and allowing the characterization of major phylogenetic groups (Selander and Levin, 1980; Tenaillon et al., 2010). Hence, each host has only one phylogroup. The 116 *E. coli* strains isolated in this study provide a representative phylogroup distribution in this group of individuals.

The phylogroup frequency in human commensal *E. coli* strains was first described by Duriez et al. (2001), whereby the most common groups were A and B1, while B2 was the rarest. However, a later comparison of human commensal *E. coli* phylogenetic groups by Bailey et al. (2010a) coincided with the results from our study, indicating a predominance of group A and an underrepresentation of group B1.

Here, we observed a higher frequency of groups A and D in wastewater samples, whereas in previous studies either groups B2 or D (Anastasi et al., 2010; Mokracka et al., 2011), or groups A and B1 (Figueira et al., 2011) were predominant. This apparent discrepancy might imply that geographic location and climate play a role in determining phylogroup distribution, since environmental strains seem to be more susceptible to these factors. Some authors have suggested group A to be the best adapted to different environments (Skurnik et al., 2008; Anastasi et al., 2012). The phylogroup distribution we observed in wastewater samples from Brazil follows those reported in Portugal (Figueira et al., 2011) and Spain (Sabaté et al., 2008), but not in Australia (Anastasi et al., 2010) or the United States (Boczek et al., 2006).

Bacterial isolates from sewage samples can be used in lieu of human feces samples (USEPA, 2005). Our results showed a positive correlation between phylogroup distributions in human and wastewater samples, reinforcing the use of isolates from urban wastewater treatment plants as surrogates for human fecal contamination. Conversely, *E. coli* commensal isolates collected in Australia, Portugal, Spain, and the United States did not show significant correlation with phylogroup distribution in isolates from WTPs samples. Contrary to these other studies, our feces and wastewater samples were collected from the same area (São Paulo Metropolitan region) and at the same time. Thus, it is possible that phylogroup frequencies fluctuate over time limiting the use of sewage samples as proxies for human ones.

Duriez et al. (2001) proposed that studies of *E. coli* commensal strains should include geographic, socioeconomic, and medical information since these enteric isolates were likely reservoirs of pathogenic strains. To this end, we compiled data from the literature that used the Clermont classification (Clermont et al., 2000), to find out if any phylogroup pattern might be related to climate, feeding habits, living area, and/or geographic location. Several studies compared Clermont phylogroups with climate (Escobar-Páramo et al., 2004; Gordon et al., 2005; Li et al., 2010), living area (Nowrouzian et al., 2005, 2009; Karami, 2007; Pallechi et al., 2007; Vinue et al., 2008; Bartoloni et al., 2009; Hannah et al., 2009; Bailey et al., 2010b; Nicolas-Chanoine et al., 2013), and feeding habits (Gordon et al., 2005), however only qualitative differences had been reported and none of the studies had formally tested the observed differences.

Here, we compared 38 samples from 24 countries and observed that groups A and B1 did not present overlapping frequencies, as opposed to groups B2 and D (Figure 2). Moreover, we report a negative correlation between phylogroup A and B2 (Figure 3). Gordon et al. (2008) studied *E. coli* strains from different hosts (humans, other mammals, and birds) and environmental samples (water, soil, and sediments) from Europe, Africa, America, and Oceania and demonstrated the validity of Clermont's method for population studies due to its rapidity, low cost, and reliability for assigning *E. coli* strains to phylogenetic groups.

The differences between phylogroup frequencies on a worldwide scale did not correlate with geographical distance, not even in the case of Mali, which presented significant differences compared to several countries on other continents. Random location clustering was also observed in the dendrogram generated by Euclidean distance, corroborating the fact that phylogroup distribution did not correlate with geographic distance. Correspondence analysis resulted in three clusters, however, no clear pattern could be identified in terms of geographic location, climate, feeding habits, or living area. Based on the successful use of the *w-clique* metric by Stoppe et al. (2014) for differentiating between polluted and unpolluted sites in river samples, we used the same principle to cluster phylogroup distributions. Once again, the obtained clusters showed no grouping by geographic proximity or climate.

Data mining results showed weak tree classification. This might occur due to the algorithm used in constructing the tree, which automatically selected only part of the data (some phylogroups). Hence, a misclassification error higher than expected may be generated when a phylogroup that is not representative of the entire sample space is selected.

The Mantel test is widely used in ecological studies to correlate genetic markers and geographic distance (Bellay et al., 2011; Castillo-Rojas et al., 2013; Winter et al., 2013). Notwithstanding, our meta-analysis data confirmed only a weak correlation between geographic location and phylogroup distribution, suggesting that the distance between sites might not influence phylogroup distribution. Some authors proposed that the human hosts' climate influenced the *E. coli* phylogroup distribution (Escobar-Páramo et al., 2004; Gordon et al., 2005; Li et al., 2010). Our analysis showed a moderate correlation between climate and phylogroup distribution, suggesting this factor can have more influence on phylogroup distribution than geographic distance.

A meta-analysis comparison using clustering multivariate analysis failed to reveal any substructuring according to the parameters evaluated, even in the cases of geographic distance and climate, which have more levels of classification (Figures 9–12). In terms of feeding habits (Figure 9), it was possible to observe three different clusters; however, each cluster comprised either western habits or other diets. In the case of living area, only one cluster was observed that contained only urban areas, although not all of them (Figure 10).

The similarity matrix according to Koppen climate classification revealed two clusters. The first one (identifier 35–37) encompassed only arid (B) and continental (D) climates, yet, some sites with these climate types were not included in the cluster. The other cluster included cities with different climates and no obvious pattern (Table 1 and Figure 11).

When looking for patterns of geographic proximity, we observed two clusters (Figure 12). The first one included cities located in North America, while the second included only cities in Europe. These results suggest that geographic location could influence phylogroup distribution. The lack of additional clusters indicates that other factors might also be at play.

Only a few studies on microbial biogeography of commensal microorganisms and their hosts have been published to date. According to Tenaillon et al. (2010) socioeconomic factors and hygiene are important determinants of phylogroup distribution, as may be diet. In developed countries, the consumption of industrialized food has increased over the past decades, whereas in developing countries the consumption of carbohydrates and fresh food are more common. This may account for the shift from A as the predominant group in developed countries and in France in 1980, to B2 being the main group in France in 2000. Nevertheless, other factors should be taken into account when determining the phylogroup distribution. In Asia, where high levels of carbohydrates are consumed, phylogroup prevalence has shifted from A (Kyoto, Japan and Seoul, Korea) (Kanamaru et al., 2006; Lee et al., 2010) to B2 (Beijing, China and Tokyo, Japan) or D (Jeonnam Province, Korea) (Unno et al., 2009). These observations suggest that neither socioeconomic factors nor diet alone is sufficient to determine phylogroup distribution patterns. Among the other variables taken in consideration in this study, none significantly affected phylogroup distribution.

Our spatial autocorrelation analysis did not reveal any specific phylogroup patterns. There is evidence of phylogroup distribution substructuring, but geographic distance does not limit *E. coli* dispersion. For free-living microorganisms, dispersion over large distances is extremely rare, but for commensal microbes it can be much easier because they can travel with their hosts. The relationship between phylogroup and their hosts is not fully understood and it must involve many other factors, besides host climate living area or feeding habits. Further research is warranted as the existence of patterns relating phylogroup distribution with host-associated factors could facilitate prediction of which *E. coli* strain is prevalent in different climate or geographic locations. Accordingly, it might act as a proxy for laboratory analysis and simplify the identification of potential pathogenic strains.

## Author contributions

NS, LO, MS, and TT: Conceived and designed the work; NS and CC: Performed the experiments; NS, JS, and TT: Analyzed the data, prepare the figures, and wrote the manuscript; MS, AS, LO, and TT: Ensured the financial and material resources; NS, JS, CC, MS, AS, LO, and TT: Reviewed the final draft.

### Conflict of interest statement

The authors declare that the research was conducted in the absence of any commercial or financial relationships that could be construed as a potential conflict of interest.
